# Construction and application of pancreatic exocrine organoid and spheroid for drug screening and precision medicine

**DOI:** 10.3389/fcell.2025.1746622

**Published:** 2026-01-06

**Authors:** Xin Tan, Bangwei Huang, Xinyi Yang, Pengyuan Wang, Lianghao Hu

**Affiliations:** 1 Department of Gastroenterology, Changhai Hospital, Naval Medical University, Shanghai, China; 2 Shanghai Institute of Pancreatic Diseases, Shanghai, China; 3 National Key Labotatory of Immunity and Inflammation, Naval Medical University, Shanghai, China; 4 Department of Gastroenterology, No. 981 Hospital of the PLA, Chengde, Hebei, China

**Keywords:** drugscreening, pancreatic cancer, pancreatic exocrine organoids, precision medicine, spheroids

## Abstract

The incidence of pancreatic exocrine disorders, particularly pancreatic cancer, has been steadily rising. However, treatment options remain limited, with substantial interindividual variability in therapeutic efficacy. This clinical challenge has accelerated the development of advanced three-dimensional (3D) modeling systems, with patient-derived organoids and multicellular spheroids emerging as transformative tools that faithfully recapitulate tumor pathophysiology. In contrast to 2D cultures, which fail to recapitulate the three-dimensional spatial architecture and cell-cell interactions found *in vivo*, these models have gained prominence in pancreatic cancer research due to their unique capacity to: (1) precisely mimic the tumor microenvironment (TME), (2) preserve tumor heterogeneity, and (3) enable rapid establishment. This review systematically examines current methodologies for constructing pancreatic exocrine 3D models, their integration with bioengineering platforms for drug screening, and innovative applications in multi-omics-driven precision medicine. We further evaluate the translational potential of these systems in clinical decision-making and discuss how they may reshape therapeutic paradigms for pancreatic diseases, offering new avenues for personalized treatment strategies.

## 1 Introduction

The pancreas is a vital organ with dual exocrine and endocrine functions, secreting digestive enzymes into the duodenum through acinar and ductal cells while regulating blood glucose. In benign exocrine disorders like chronic pancreatitis, progressive parenchymal injury and interstitial fibrosis leads to maldigestion, steatorrhea, and malnutrition, with no available therapies to delay or reverse pancreatic fibrosis (Giustarini et al., 2023; Hart and Conwell, 2020; Malik et al., 2023). For pancreatic ductal adenocarcinoma (PDAC), the most aggressive pancreatic malignancy with a 5-year survival rate of only 11%, most patients present with metastatic unresectable disease, gemcitabine-based chemotherapy shows merely 20% efficacy due to resistance mechanisms, and emerging immunotherapies face challenges from interpatient heterogeneity (Eso et al., 2020; Gu et al., 2021; Siegel et al., 2022). These clinical unmet needs underscore the critical importance of developing pathologically relevant models to advance both pancreatic disease therapeutics and precision oncology approaches.

Organoids are miniature 3D organ models that self-organize from adult stem cells (ASCs), embryonic stem cells (ESCs), or induced pluripotent stem cells (iPSCs) faithfully replicating both the structure and function of native tissues (Corrò et al., 2020). Particularly valuable are patient-derived organoids (PDOs), which maintain tumor heterogeneity and accurately mirror the histological, genomic, and proteomic profiles of original tumors - making them ideal for personalized medicine (Boj et al., 2015). When co-cultured with cancer-associated fibroblasts (CAFs) or immune cells, PDOs can better recapitulate the TME, significantly improving clinical predictability (Schuth et al., 2022; Tsai et al., 2018). In contrast, spheroids are simpler 3D cell aggregates (from primary cells or cell lines) that mimic basic cell-cell and cell-matrix interactions (Ryu et al., 2019). Current pancreatic spheroid systems incorporate multiple cell types such as pancretic progenitor cells, ductal cells, acinar cells, pancreatic stellate cells (PSCs), tumor cells, and immune cells (Giustarini et al., 2023; Grapin-Botton, 2016). While spheroids offer advantages like easier culture, faster generation, and lower cost, they have limited capability to mimic the *in vivo* environment compared to organoids (van Renterghem et al., 2023). The integration of 3D cell culture with microfluidics, AI, automation, and computational tools has enabled high-throughput production, intelligent monitoring, and standardized evaluation - advancing its application in drug screening (Ma et al., 2021; Wang and Jeon, 2022). Furthermore, combining patient-derived pancreatic organoids with multi-omics analysis has deepened our understanding of PDAC tumor heterogeneity and molecular profiles, facilitating the development of personalized therapies targeting specific molecular features to improve clinical outcomes (Boilève et al., 2024). This review summarizes recent progress in pancreatic organoid and spheroid technologies for drug screening and precision medicine, highlighting how engineered platforms and multi-omics approaches are driving these advancements.

## 2 Establishment of pancreatic organoids and spheroids

An important breakthrough in pancreatic organoid research originated from the discovery by Meritxell Huch’s team (Huch et al., 2013): *in vitro* Lgr5+ pancreatic ductal cells exhibit stem cell characteristics, capable of differentiating into both endocrine and exocrine pancreatic cells, while maintaining genetic stability and proliferative capacity over long-term culture exceeding 10 months. Based on this stable *in vitro* culture system, organoids can not only accurately replicate the developmental dynamics, three-dimensional structure, and physiological functions of pancreatic organs but also construct disease models using gene editing technologies (Kim et al., 2020). Currently, modeling of pancreatic exocrine organoids mainly comes from ASCs, iPSCs, or ESCs, in which organoids derived from ASCs maintain stable and continuous passage while reflecting the characteristics of their source tissues, making them suitable for ongoing drug screening and personalized disease research; organoids derived from iPSCs or ESCs can simulate pancreatic development during embryonic development, facilitating the study of disease mechanisms or early interventions in disease progression (Grapin-Botton, 2016). In this section, we will discuss the construction methods of pancreatic exocrine organoids and the challenges currently faced.

### 2.1 ASCs-derived pancreatic organoids

**FIGURE 1:**
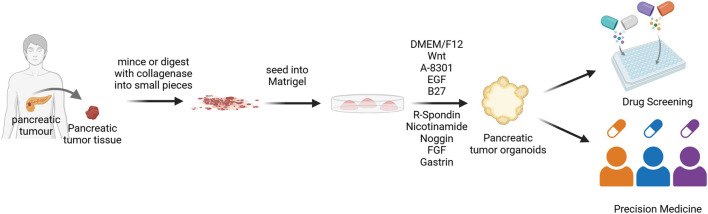
Flowchart of PDOs construction. Tumor tissues obtained from patients are digested using enzymatic digestion methods, then seeded into Matrigel to differentiate into pancreatic exocrine organoids (Rauth et al., 2021). DMEM/F12, Dulbecco’s Modified Eagle Medium/Nutrient Mixture F-12; EGF, Epidermal growth factor; FGF, Fibroblast growth factor.

ASCs that can differentiate into both endocrine and exocrine pancreatic cells exist within the pancreatic ducts, and during pancreatic injury, they can re-express the endodermal lineage marker PDX1 through a dedifferentiation process, thereby recreating the developmental differentiation pathway (Li et al., 2010; Xu et al., 2008). Building on this ductal cell potential, pancreatic tissues undergo collagenase digestion and filtration to isolate ductal fragments. These fragments are then seeded into Matrigel, where they self-organize into pancreatic organoids containing functionally distinct endocrine and exocrine cell populations (Huch et al., 2013; Seino et al., 2018) (Figure 1). Adult stem cell-derived pancreatic organoids primarily focus on pancreatic tumor organoids, with two common construction methods: one is to directly digest pancreatic tumor tissue obtained from surgical resection or fine needle aspiration biopsy, achieving a success rate of 85% (Huang et al., 2015); the other is genetic modification of normal pancreatic organoids via CRISPR-Cas9-mediated oncogene editing or transcriptional reprogramming, with a success rate of about 70% (Kawasaki et al., 2020). As the most clinically relevant *in vitro* models, patient-derived organoids (PDOs) from ASCs faithfully recapitulate tumor heterogeneity and histopathological features, making them invaluable for personalized medicine (Dayton et al., 2023; Tong et al., 2024). Notably, pancreatic cancer PDOs demonstrate 91.1% predictive accuracy for patient responses to first-line chemotherapies (Beutel et al., 2021). Although ASC-derived pancreatic organoid technology has been widely applied in pancreatic cancer research, the development of organoid models for benign inflammatory diseases, such as chronic pancreatitis, remains in its early stages (Chen et al., 2024) This is primarily due to several technical bottlenecks. First, obtaining clinical tissue samples from benign lesions is challenging. Second, cultured cells from chronic pancreatitis, such as acinar cells, are prone to spontaneous dedifferentiation *in vitro*, making it difficult to maintain their functional phenotypes long-term Finally, a core feature of the disease—the sustained inflammatory and fibrotic microenvironment—involves complex interactions among immune cells, stellate cells, and the extracellular matrix. Recapitulating this dynamic process *in vitro* represents the most significant current challenge. Notably, this spontaneous dedifferentiation observed in culture models the key pathological process of “acinar-to-ductal metaplasia,” which links pancreatitis to preneoplastic lesions, providing a unique entry point for disease modeling (Zuiani et al., 2024).

The culture medium and matrix gel are critical determinants of organoid culture. The matrix gel, typically derived from Engelbreth-Holm-Swarm (EHS) mouse tumor extracts, provides a basement membrane scaffold enriched with extracellular matrix proteins (Kleinman and Martin, 2005). For pancreatic organoid culture, a growth factor-reduced formulation is preferred, as the native matrix gel contains over 1,800 proteins that could confound cellular behavior, signaling pathways, and cytokine analyses (Boj et al., 2015; Hughes et al., 2010; Raghavan et al., 2021). On the other hand, the culture medium must precisely modulate key signaling pathways to enable organoid self-assembly: Wnt/β-catenin activators (RSPO1, Wnt3a, Noggin) stimulate ASCs via Lgr5 (Huch et al., 2013; Yan et al., 2017); EGF and FGF10 sustain pancreatic progenitor proliferation (Ndlovu et al., 2018); while EGF with nicotinamide enhances ductal cell clonogenicity (Wedeken et al., 2017); Supplements like B27 further support organoid morphogenesis. However, mitogenic factors present a double-edged sword: EGF/FGF not only drive proliferation but also induce scBasal-to-scClassical transition in PDAC organoids, altering transcriptional profiles (Raghavan et al., 2021), and may promote acinar-ductal metaplasia (Perera and Bardeesy, 2012). This necessitates careful optimization of culture conditions to balance organoid expansion with preservation of native tissue characteristics.

### 2.2 iPSCs-derived or ESCs-derived pancreatic organoids

**FIGURE 2:**
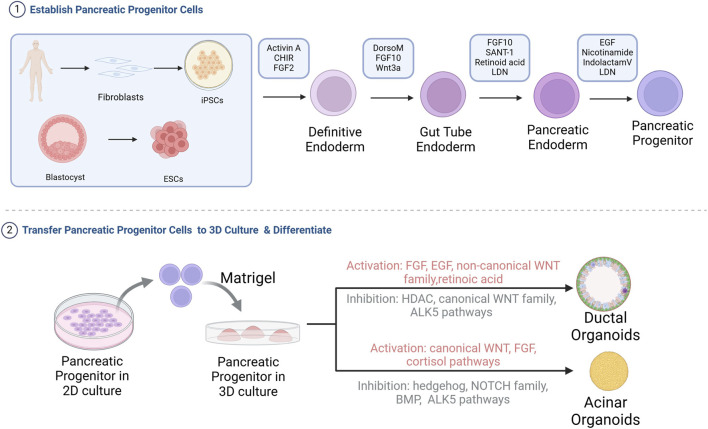
Flowchart of the construction of pancreatic acinar or ductal organoids from iPSCs or ESCs. iPSCs or ESCs are differentiated into pancreatic progenitor cells in 2D culture, followed by seeding into Matrigel for directed differentiation into pancreatic acinar or ductal organoids. HDAC, Histone deacetylases; BMP, Bone morphogenetic protein (Breunig et al., 2021; Huang et al., 2021; Pagliuca et al., 2014; Wiedenmann et al., 2021).

Unlike ASCs in pancreatic tissue, which can only differentiate into pancreatic cells, ESCs or iPSCs have a higher differentiation potential and can be induced into almost all cell types in the body (Tian et al., 2023). The developmental pathway for pancreatic organoids from these pluripotent stem cells recapitulates embryonic pancreas development. This process begins with differentiation of ESCs/iPSCs into definitive endoderm, progresses through primitive gut tube formation, and finally develops into multipotent pancreatic progenitor cells (MPCs) expressing key markers Ptf1a and Pdx1 (Jennings et al., 2015; Pan and Wright, 2011). To mimic this developmental sequence *in vitro*, specific signaling pathways are sequentially modulated using defined growth factors and small molecules, as summarized in Table 1. Next, these pancreatic progenitor cells are then embedded in Matrigel for 3D organoid culture (Figure 2). Further purification using cell sorting techniques to isolate glycoprotein-2 positive (GP2+) progenitor populations enhances their ability to differentiate into all three pancreatic lineages - acinar, ductal, and endocrine cells - enabling generation of corresponding organoid types (Merz et al., 2023).

**TABLE 1:** Summary of key compounds for inducing iPSC/ESC-derived pancreatic organoids.

Stage	Differentiation stage/target	Targeted pathway/mechanism	Compounds
Pancreatic development stages (Jennings et al., 2015; Pan and Wright, 2011)	Definitive endoderm formation	Activates TGF-β signaling	GDF8, Activin A (Wei and Wang, 2018)
	Primitive gut tube formation	Provides FGF signaling	FGF7, FGF10
	Pancreatic progenitor specification	Retinoic acid signaling; inhibits Hedgehog; inhibits BMP	Retinoic Acid, SANT1, LDN193189 (Pagliuca et al., 2014; Rezania et al., 2014)
Lineage specification stages	Acinar organoids	Sustained activation of Wnt/β-catenin	CHIR, SKL2001, WNT1 (Keefe et al., 2012; Liu et al., 2022; Wells et al., 2007; Yu et al., 2019)
		Induces acinar gene expression	Dexamethasone (Logsdon et al., 1985)
		Inhibits Hedgehog pathway; Inhibits Hippo pathway:prevents ductal fate	HPI-1,XMU-MP-1 (Fan et al., 2016; Hyman et al., 2009; Pasca di Magliano et al., 2006; Wu et al., 2021)
		Biphasic modulation of Notch pathway: Early proliferation vs. late maturation	Early: FGF10; Late: Dibenzazepine (Hald et al., 2003; Hart et al., 2003)
	Ductal organoids	Inhibits canonical Wnt signaling	IWP2, iCRT14, IQ1 (Bilir et al., 2013; Haumaitre et al., 2008)
		Activates non-canonical Wnt signaling	Foxy5 (Leszczynska et al., 2024; Sasaki and Kahn, 2014)
		Inhibits histone deacetylase	SB939, WT161 (Shen et al., 2022; Sun et al., 2021; Wang et al., 2023)
	Commonly used (early stage)	Progenitor maintenance/Apoptosis inhibition	FGF10, Y27632, EGF (Ndlovu et al., 2018; Norgaard et al., 2003)

The ability to derive both acinar and ductal organoids from pluripotent stem cells provides powerful tools for modeling pancreatic disease development. Ling Huang’s group established a four-stage differentiation protocol to generate these organoid types from pancreatic progenitor cells (Huang et al., 2021). From iPSC to pancreatic organoids, the required compounds and their roles are listed in Table 1. During pancreatic organoid differentiation, progenitor markers (NKX6.1, Pdx1) gradually decrease while lineage-specific markers emerge. Mature organoids exhibit characteristic polarization, with tight junction protein ZO-1 localized apically and basement membrane components (collagen IV, laminin-α5) basally (Hohwieler et al., 2017; Huang et al., 2021). Acinar and ductal organoids differ markedly in morphology, marker expression and functional characteristics (summarized in Table 2). A key limitation is that current differentiation protocols typically produce organoids resembling fetal rather than adult pancreatic tissue (Huang et al., 2015). This developmental immaturity likely reflects the absence of crucial niche interactions present during normal pancreatic development that are difficult to replicate *in vitro*. Overcoming this limitation represents an important challenge for the field.

**TABLE 2:** Comparison of acinar and ductal organoids.

Organoid type	Structure	Function	Markers	References
Acinar organoids	Small diameter, 20–105 μm, no lumen	High amylase and lipase activity	PTF1A, HNF1B chymotrypsin C, RBPJL, CPA1, amylase	Hohwieler et al. (2017), Huang et al. (2021)
Ductal organoids	Large diameter, 50–220 μm, visible lumen expansion	Functional CFTR; high carbonic anhydrase activity	SOX9, carbonic anhydrase II, CK19	Hohwieler et al. (2017), Huang et al. (2021)

Compared to ASC-derived organoids, those generated from ESCs or iPSCs offer unique advantages in modeling developmental processes and disease progression. These systems enable researchers to trace the evolutionary trajectory of pathogenic mutations through gene editing technologies, providing valuable platforms for personalized medicine (Breunig et al., 2021; Hirshorn et al., 2021). Huang et al. (2015) demonstrated this potential by establishing KRAS- and TP53-mutated pancreatic cancer organoids from human pluripotent stem cells. Their 3D culture system not only revealed molecular mechanisms of pancreatic carcinogenesis but also identified genotype-phenotype correlations and enabled drug screening for personalized treatment strategies. For benign diseases, iPSC-derived pancreatic ductal organoids harboring the CFTR ΔF508 mutation exhibited no swelling response upon forskolin treatment, indicating a chloride ion transport defect. These organoids can be used to screen CFTR modulators, offering a potential strategy to guide personalized therapy for patients with cystic fibrosis (Mun et al., 2022).

### 2.3 Pancreatic spheroids

Compared to conventional monolayer cultures, 3D spheroids demonstrate significantly increased resistance to chemotherapy. The concentric layered structure of spheroids creates a physical barrier where outer cells protect inner cells from drug penetration, mimicking the drug diffusion limitations seen in solid tumors *in vivo* (Perche and Torchilin, 2012). Notably, PDAC spheroids show a 200-fold increase in IC50 values for gemcitabine and oxaliplatin compared to their 2D counterparts (Firuzi et al., 2019). Furthermore, the 3D architecture more accurately reproduces the complex cell-cell and cell- extracellular matrix (ECM) interactions found in native tumor tissue (Ishiguro et al., 2017). Moreover, spheroid culture enables precise control over spheroid size, enhances data reproducibility, and facilitates high-throughput screening when integrated with advanced technologies like 3D printing. These advantages make spheroid models an ideal preclinical platform for drug screening (Friedrich et al., 2009; Huang et al., 2023). Pancreatic spheroids are primarily generated from established 2D cell lines using scaffold-based or scaffold-free methods. Scaffold-based cultures utilize agarose, collagen, or gelatin to mimic the ECM, facilitating cell aggregation, proliferation, and migration. In contrast, scaffold-free approaches employ ultra-low adhesion surfaces, hanging drop techniques, or microfluidics to promote spontaneous spheroid formation (Gündel et al., 2021). Beyond cell lines, patient-derived tumor cells can also form spheroids. For instance, microfluidic-assisted 3D models encapsulate primary pancreatic cancer cells, generating stable tumor spheroids that retain patient-specific heterogeneity and enable drug efficacy assessment (Song et al., 2023). Compared to organoids, spheroids offer simpler handling, faster culture, and lower cost. However, their ability to recapitulate the *in vivo* microenvironment remains inferior to both organoids and animal models (Białkowska et al., 2020).

### 2.4 Pancreatic 3D co-culture model

Monotypic tumor organoids and spheroids lack essential stromal constituents-including CAFs and immune infiltrating cells, and therefore fail to faithfully reproduce the complexity of the native tumor microenvironment. Specifically, without CAFs, the models cannot reproduce their promotion of tumor-cell proliferation, motility, and therapy resistance, nor their capacity to dampen immune surveillance. Similarly, the omission of immune cells can markedly distort organoid-based predictions of responses to immunotherapies, chemotherapeutics, and radiotherapy (Guinn et al., 2024; Peng and Oberstein, 2025). To better recapitulate the intricate cellular interplay of the pancreatic TME, investigators have established multiple coculture platforms integrating pancreatic organoids/spheroids with stromal cell counterparts (Hwang et al., 2019; Tsai et al., 2018). Table 3 provides a comparative summary of spheroid coculture approaches across studies, including system architecture, cell-type composition, and coculture ratios.

**TABLE 3:** Construction of co-culture spheroid systems in pancreatic cancer modeling.

Spheroid type	Modeling Features	Cell types and ratio	Advantages	References
Spheroid-on-chip	• Collagen type I (Col I) hydrogel • Microfluidic platform	750 cells/device Pancreatic cancer cell lines (BxPC-3, Capan-2, Panc-1, MIA PaCa-2):human pancreatic stellate cells = 2:1	Enables study of the effects of fluid shear stress, matrix stiffness, and other biophysical cues on tumor growth	Hernández-Hatibi et al. (2025)
Spheroid	• Ultra-low attachment plates • Use of Orbits™ software to quantify cell ratios; the mixing ratio of the three target cell types was iteratively optimized by comparing the cellular composition of cultured spheroids with that of PDAC tumors	3000–7500 cells/spheroid (1) BxPC-3: RLT-PSC: HMEC-1 = 7 : 2: 4 (2) BxPC-3: hPSC21: HMEC-1 = 6 : 5: 3 (3) MiaPaCa-2: RLT-PSC: HMEC-1 = 6 : 3: 3 (4) MiaPaCa-2: hPSC21: HMEC-1 = 5 : 6: 4	1. Effectively recapitulates key features of the PDAC tumor microenvironment 2. Demonstrates that tumor cell drug resistance is influenced by cellular composition, density, and spatial organization	Verloy et al. (2025)
Multi-layer spheroid	• Ultra-low attachment plates • Fibrotic-core multilayer spheroid without exogenous matrix. The core is a compacted mixture of tumor cells and CAFs, followed by an outer layer of additional CAFs, constrained by an outermost alginate hydrogel shell	Tumor cells: CAFs = 1 : 1 (250 cells: 250 cells) for the core mixture; the outer layer is formed by adding 4000 CAFs	1. Structurally stable for up to 14 days 2. Exhibits higher drug resistance, faster tumor growth rate, and increased expression of immunosuppressive cytokines	Jang et al. (2024)
Spheroid	Ultra-low attachment plates	35,000 or 40,000 cells/well Pancreatic cancer cells (MiaPaCa2): Immune cells (U937 or CD11b^+^ cells) = 1 : 3 to 1 : 6	Facilitates the design of novel neutrophil-based immunotherapy strategies	Shao et al. (2024)

Analogously, organoid coculture models incorporating essential TME stromal constituents markedly improve biological fidelity and predictive performance. These systems fall into two major categories: (1) coculture with CAFs to model CAF-driven inflammatory activation and epithelial-mesenchymal transition (EMT) reprogramming, leading to diminished chemotherapeutic sensitivity (Gout et al., 2025); while (2) coculture with immune cells reconstitutes the immune microenvironment, enabling robust preclinical assessment of immune-checkpoint blockade, CAR-T therapy, and other immunotherapeutic modalities (Meng et al., 2021; Zhou et al., 2023). Expanding these systems to multicellular cocultures incorporating immune cells, CAFs, and tumor cells enables detailed dissection of TME interaction networks and aids in the discovery of targeted interventions (Tsai et al., 2018). Current co-culture methods primarily include (1) direct contact approaches, where dissociated organoids and co-cultured cells are embedded together in Matrigel to allow cell-cell interactions, and (2) indirect contact systems, which use transwell chambers to physically separate 3D-cultured cells in the lower chamber from co-cultured cells in the upper chamber (Öhlund et al., 2017).In conclusion, by incorporating essential TME elements, these next-generation models markedly enhance preclinical predictive power and furnish critical technological foundations for innovative therapy development.

### 2.5 Pancreatic 3D culture on chip

Although fibroblasts or immune cells have been incorporated into coculture systems, the lack of vascularization and static perfusion within the TME persists, limiting their capacity to fully recapitulate the complexity of the human tumor microenvironment. These limitations include reduced viability due to insufficient oxygenation, phenotypic drift, and inaccurate drug-response predictions. In recent years, integrating pancreatic organoids/spheroids with microfluidic chip technology has provided a promising solution to these challenges. These microfluidic platforms incorporate perfusable microvascular networks that support the growth, maturation, and function of 3D models, enabling highly biomimetic and dynamic TME simulations (Quintard et al., 2024). The advantages of microfluidic platforms are summarized below: First, the perfusable vascular system provides tumor and immune cells with appropriate ECM architecture and mechanical microenvironments, allowing more accurate support of immune-cell infiltration, activation, and intercellular communication, thereby offering significant advantages for evaluating immunotherapies (Choi et al., 2024). Second, this system enables dynamic, drug transport kinetics and therapeutic efficacy (Kim et al., 2025). When integrated with automated control, the system enables precise regulation of nutrient and drug delivery and supports high-throughput, parallel testing of multiple drug sensitivities, significantly improving screening efficiency and accuracy (Castiaux et al., 2019; Schuster et al., 2020). Furthermore, organ-on-chip technology offers the potential for multi-organ integration. By linking pancreas-specific chips with other organ systems, a “human-on-a-chip” platform can be constructed to investigate inter-organ communication, systemic metabolic pathways, and whole-body toxicity (Wang et al., 2019). In summary, microfluidic technology provides a powerful platform for studying the tumor microenvironment, drug responses, and immune interventions *in vitro* by constructing perfusable vascular networks, enabling precise environmental control, and incorporating automated operations.

Following the detailed description of the four major 3D models, a comprehensive comparison of their key characteristics is provided in Table 4, summarizing their respective culture systems, tumor microenvironment recapitulation, genetic stability, and strengths/limitations in drug screening.

**TABLE 4:** Comparison of pancreatic exocrine gland 3D models.

3D models	Culture system	Recapitulation of TME	Genetic stability	Drug screening/prediction strengths	Drug screening/prediction limitations
ASCs-derived pancreatic organoids	Require hydrogel and multiple growth factors to promote progenitor cell expansion, differentiation, and self-assembly into organoids	Can form a personalized tumor microenvironment through co-culture with patient-matched immune or stromal cells (Tsai et al., 2018; Tsai et al., 2018)	Key genetic mutations, transcriptomic profiles, histological architecture, and intratumoral heterogeneity of the primary tumor can be maintained long-term (Sljukic et al., 2025)	(1) Capable of long-term, stable expansion (2) Multi-cellular characteristics recapitulate the complexity of the original tumor	(1)High cost (2) Dependent on biopsy; difficult to obtain samples from benign diseases (3) Lack of vascularization
iPSCs-derived or ESCs-derived pancreatic organoids	Following *in vitro* induction to pancreatic progenitor cells, directed differentiation into specific lineages occurs in hydrogel with specific growth factors; the culture medium composition is more complex	It holds the potential to differentiate into multiple lineages from an isogenic origin, allowing for the study of tumor microenvironments shaped by specific genetic backgrounds. (Takeuchi et al., 2025)	The reprogramming process may introduce genetic mutations (Yan et al., 2023)	(1) Solves the problem of difficult tissue sourcing for benign diseases (Hohwieler et al., 2017) (2) Enables study of disease pathogenesis in specific genetic contexts, allowing for the investigation of early intervention strategies (Merkle et al., 2021)	(1) Complex, time-consuming, and costly construction process (2) Lack of acquired cancer gene mutations (Yan et al., 2023) (3) Incomplete simulation of pancreatic maturity (Huang et al., 2021)
Pancreatic spheroids	Spheroids are constructed by scaffold-based or scaffold-free methods. The culture medium is relatively simple, primarily supporting basic cell growth and aggregation. (Jamshidi et al., 2025)	(1) Simulates the physical barrier through tumor cell-stromal cell interactions (2) Recapitulates the intratumoral nutrient gradient and hypoxic core	easily lost during long-term culture	(1) Short construction time, suitable for high-throughput drug screening (2) Simulates drug diffusion gradient (3) Can simulate cell-cell interactions	(1) Short spheroid survival time (2) Requires a large number of cells
Pancreatic 3D culture on chip	Mature organoids/spheroids are placed in chip chambers, preserving their 3D structure and cell polarity	(1) Simulates tumor-induced angiogenesis (2) Allows precise control over multiple microenvironmental factors (Li et al., 2023) (3) Enables 3D interactions among tumor cells, stroma, vasculature, and immune components	Depends on the type of 3D cultured cells placed on the chip	(1) Microfluidic models require minimal cells (2) Enables real-time monitoring of 3D culture dynamics (Ruan et al., 2025) (3)Accelerates organoid cell differentiation and maturation (4) Allows gradient drug administration, simulating plasma concentration gradients (Shen et al., 2020)	(1) High technical difficulty and cost (2) Lacks unified technical evaluation standards

## 3 Pancreatic organoids and spheroids for drug screening and precision medicine

The current applications of pancreatic 3D organoid or spheroid culture are primarily in pancreatic cancer. Organoids have shown 100% sensitivity and 93% specificity in predicting drug response, indicating their significant potential for high-throughput drug screening and precision medicine (Vlachogiannis et al., 2018). Spheroid models exhibit higher chemical resistance when responding to drug efficacy, simulating the TME and providing greater predictive value for drug effectiveness (Jamshidi et al., 2025). With the development of various 3D model construction methods and the integration of omics technologies, organoids and spheroids will continue to advance pancreatic cancer research and treatment.

### 3.1 High-throughput drug screening

The current translational success rate of drug development is only 5%, resulting in significant resource waste; the main reason is that existing models primarily rely on 2D cell cultures and animal models, the former of which fails to simulate the *in vivo* environment and reflect disease heterogeneity, while the latter suffers from species differences, resulting in a lower success rate in drug development (Brancato et al., 2020). While patient-derived xenografts (PDXs) faithfully maintain tumor heterogeneity and remain valuable for preclinical studies, their utility is constrained by prolonged establishment times (typically 4–6 months), high maintenance costs, and limited throughput capacity (Lau et al., 2020). In contrast, 3D culture systems offer several distinct advantages: (1) They enable high-throughput screening in standardized 96- or 384-well formats with establishment times of just days to weeks (Hung et al., 2024; Longati et al., 2013; Wong et al., 2019). (2) They permit precise control over the tumor microenvironment, including adjustable extracellular matrix composition, stromal cell populations, and biophysical gradients that are difficult to manipulate in PDXs (Debruyne et al., 2024); (3) They better preserve human tumor metabolism without murine contamination while allowing real-time metabolic monitoring (Griffin, 2006; Longati et al., 2013). and (4) They rapidly recapitulate critical tumor phenotypes like chemoresistance and invasion - for instance, pancreatic cancer cell migration increases threefold when co-cultured with CAFs in 3D models, with drug resistance patterns emerging within weeks rather than months (Krulikas et al., 2018; Wong et al., 2019); Although 3D cultures cannot fully replace PDXs for *in vivo* validation, they provide a scalable, cost-effective platform for preliminary drug screening and mechanistic studies that complements PDX models in the drug development pipeline.

Despite their advantages, current 3D models face several limitations in drug screening applications. First, heterogeneity in morphology, cell composition, and spatial organization compromises experimental reproducibility (Ma et al., 2021). Standardized bioengineering approaches (summarized in Table 5) using automated platforms are addressing this challenge by improving model consistency. Second, conventional endpoint assays (e.g., ATP measurement) lack spatiotemporal resolution for dynamic drug response assessment in 3D systems (Deben et al., 2023). High-content imaging technologies now enable real-time, multidimensional analysis while preserving 3D architecture. Researchers such as Shoko Tsukamoto have successfully utilized real-time dynamic imaging technologies to systematically monitor organoids, elucidating the heterogeneous growth characteristics of patient-derived organoids and their differential drug response patterns (Fraietta and Gasparri, 2016; Tsukamoto et al., 2025). However, the resulting data deluge presents new challenges, driving development of AI-based analytical tools, For example, Jonathan M. Matthews and others have invented software for the automated identification, tracking, and analysis of individual organoid dynamics, applied for real-time monitoring of pancreatic cancer organoids, with a tracking accuracy maintained at over 89% (Matthews et al., 2022). These converging technological advances are establishing a new paradigm for high-precision organoid drug screening.

**TABLE 5:** Bioengineering methods for constructing pancreatic 3D models for drug screening.

Model type	Construction method	Constructed cells/tissues	Application	Detection indicators	Advantages	References
Spheroid	Droplet Extrusion 3D Bioprinting (DEP)	BxPC-3 cell line, normal human dermal fibroblasts	Simulates the stromal abundance features of pancreatic cancer tissues, constructing a drug screening platform that reflects drug resistance characteristics of pancreatic cancer	Cell viability	The DEP system prints in combination with GelMA hydrogel, achieving high throughput while avoiding contamination with animal-derived components from Matrigel	Huang et al. (2023)
Spheroid	Magnetic 3D Bioprinting	primary patient-derived pancreas cancer cells and CAFs	Constructs a high-throughput drug screening platform for primary pancreatic cancer	Cell viability	The first large-scale screening effort, screening over 150,000 small molecules	Fernandez-Vega et al. (2022)
Organoids	Automated liquid handling system, CRISPR-Cas9	PC02 and PC02e cell lines	Constructs a high-throughput drug screening platform based on 3D culture and improves personalized medicine for pancreatic cancer through gene editing combined with drug screening	Cell viability	Combines drug screening with CRISPR-Cas9 genome editing to screen drugs targeting common PDAC driver gene mutations, identifying populations sensitive to targeted therapies, with high-throughput screening of 1,172 drugs	Hirt et al. (2022)
Pancreatic organoid chip	Microfluidic Platform	Constructs organoids from patient tumor tissues	Constructs an automated, high-throughput microfluidic 3D organoid culture and analysis system to facilitate preclinical research	Cell apoptosis	Supports real-time analysis of organoids, and the valve control of the microfluidic platform is conducive to high-throughput drug screening	Schuster et al. (2020)
		Panc-1 cell line, fibroblast cells	Constructs an innovative surface microfluidic platform with quadrants to assess the effects of the extracellular matrix on tumor invasiveness, as well as the responses to chemotherapeutic agents	Cell viability	Integrates 3D tumor spheroids, stromal cells, and ECM microenvironments, enabling multi-drug screening and *in situ* analysis of invasiveness, providing a precise drug screening platform	Seyfoori et al. (2025)

### 3.2 Precision medicine

Current first-line therapy for pancreatic cancer relies on cytotoxic agents such as gemcitabine; however, therapeutic efficacy is constrained by frequent drug resistance and the lack of effective targeted treatments (Knudsen et al., 2016). These therapeutic failures arise from multiple factors, including genetic heterogeneity, the complexity of the TME, immune suppression, and metabolic reprogramming (Beatty et al., 2021). Developing precision-medicine strategies tailored to individual patient characteristics has become a critical approach for overcoming current therapeutic limitations. Because patient-derived organoids (PDOs) retain the molecular and morphological features of primary tumors and allow drug screening within a short timeframe, they have become an essential platform for supporting precision-medicine research and applications. Foundational work on using PDOs to predict drug responses in pancreatic disease began in 2015, when Ling Huang and colleagues first demonstrated drug-effect assessment using pancreatic cancer organoids (Huang et al., 2015). Subsequently, in 2018, Tiriac and colleagues correlated PDO drug-sensitivity profiles with the clinical outcomes of corresponding patients, providing preliminary validation of their predictive potential (Tiriac et al., 2018). Currently, the application of PDOs in pancreatic-cancer precision medicine focuses primarily on the following areas: (1) designing targeted treatment or combination-therapy strategies based on PDO responses to various drugs to improve therapeutic outcomes; (2) elucidating the molecular mechanisms underlying tumor heterogeneity and integrating multi-omics analyses to identify personalized therapeutic targets in pancreatic cancer; (3) identifying mechanisms associated with tumor-cell drug resistance using PDOs derived from resistant patients, thereby providing a theoretical basis for overcoming resistance (Xiang et al., 2024).

### 3.3 Bridging basic research and clinical practice with pancreatic PDOs

**FIGURE 3:**
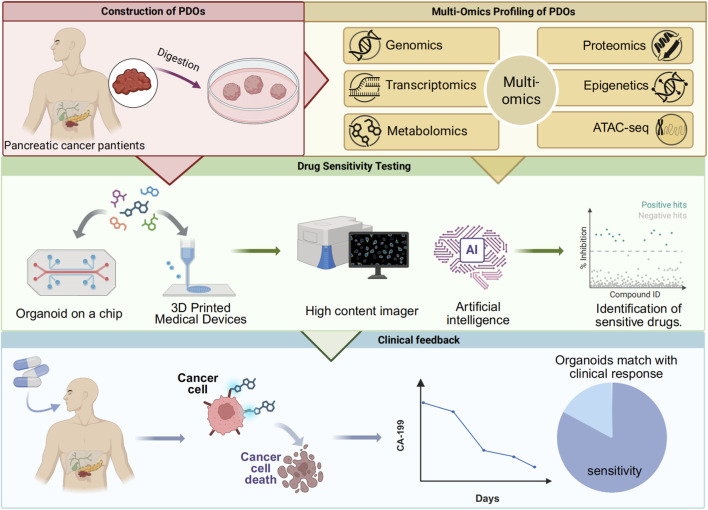
An integrated pathway of organoid establishment, multi-omics analysis, drug sensitivity testing, and clinical feedback for precision medicine.

PDO models hold significant value in the precision medicine of pancreatic cancer. Their core utility lies in establishing a complete workflow of “organoid establishment, multi-omics analysis, drug sensitivity testing, clinical feedback and validation” (Figure 3). However, crucially, the clinical implementation of this workflow is contingent upon the efficiency of its initial step: the generation and testing of PDOs must be completed within the clinical decision-making window. Although drug screening based on the genomic features of PDOs has shown potential to extend patient survival, traditional PDO generation methods are time-consuming. This prolonged timeline often misaligns with the rapidly progressive nature of pancreatic cancer, ultimately limiting patient benefit in practice (Sarno et al., 2025). Consequently, improving the time efficiency of PDO generation and testing is pivotal for advancing its clinical translation. Currently, studies have demonstrated that the PDO testing cycle can be substantially shortened through technical optimization. For instance, constructing PDOs from biopsy samples prior to neoadjuvant therapy, coupled with 3D printing technology, can reduce the median turnaround time for drug sensitivity testing to 48 days (Seppälä et al., 2020), which precedes the conventional start time for postoperative adjuvant chemotherapy (typically 8–12 weeks after surgery). Furthermore, Wu et al. successfully generated PDOs from circulating tumor cells, shortening the culture cycle to approximately 3 weeks. The drug sensitivity results showed a significant correlation with patient clinical response, providing a feasible pathway for real-time individualized therapy. However, the small sample size of this study necessitates validation of its predictive accuracy in larger cohorts (Wu et al., 2022).

Following successful organoid establishment, genetic sequencing revealed that 79.6% of organoids harbored potentially targetable genetic alterations. Targeting these mutations with combination therapies of targeted agents and conventional chemotherapeutic drugs has become a pivotal pathway for leveraging PDOs to advance precision therapy (Sarno et al., 2025). For instance, a study established a biobank of 260 pancreatic cancer PDOs. Metabolomic data indicated a significant elevation in cholesterol metabolism levels in pancreatic cancer. Statins were found to selectively inhibit the growth of chemotherapy resistant organoids *in vitro*. Based on this finding, a phase II clinical trial (NCT06241352) was conducted, demonstrating that combination therapy with statins and traditional chemotherapy reduced CA19-9 or CEA levels by more than 20% in 70.3% of patients after 1 month, with tumor volume shrinkage observed in most patients (Li et al., 2025). Furthermore, addressing the most prevalent driver gene, KRAS (mutation rate >90%), and resistance to its inhibitors (Beatty et al., 2021; Knudsen et al., 2016), research utilizing KRAS G12D-mutant PDO models through multi-omics analysis identified activation of the EMT and the PI3K-AKT-mTOR pathway as key bypass resistance mechanisms. This provides a direct rationale for designing combination treatment strategies (Dilly et al., 2024). In summary, the integration of organoid models with multi-omics analysis can uncover PDAC resistance mechanisms from multiple dimensions—such as metabolic reprogramming and signaling pathway dysregulation and identify novel therapeutic targets.

To effectively translate PDO drug sensitivity and multi-omics profiles into clinical guidance, it is imperative to advance beyond static testing toward high-content, dynamic analytical platforms. On one hand, technologies such as quantitative live-cell imaging enable real-time monitoring of drug responses at the level of single-cell signaling pathways. For example, by tracking the dynamic activities of ERK/AMPK kinases in real time, temporal dependencies of different kinases during organoid growth can be revealed, informing combination therapy strategies (Tsukamoto et al., 2025). On the other hand, intelligent imaging platforms, represented by tools like OrganoID, allow label-free, high-throughput monitoring of macroscopic dynamic changes in organoid number, size, and morphology. These platforms integrate artificial intelligence algorithms for real-time analysis, using the acquired imaging data to accurately assess drug efficacy based on organoid growth (Le Compte et al., 2023; Matthews et al., 2022). Together, these technologies are advancing drug sensitivity testing from simple endpoint cytotoxicity assessments toward multidimensional, dynamic, and precise analysis.

Building upon the aforementioned technologies, the feasibility and value of PDO-guided clinical therapy have received strong support from prospective studies. The largest prospective study to date demonstrated that treatment plans based on PDO drug sensitivity results enabled 91% of pancreatic cancer patients to receive matched personalized therapy, significantly improving progression-free survival and objective response rates, thereby substantiating its clinical utility (Boilève et al., 2024). Concurrently, efforts to standardize and normalize organoid technology are accelerating: an expert consensus published in 2024 systematically standardized the drug sensitivity testing workflow, signifying that the technology has reached a level suitable for clinical application and holds promise for reducing treatment risks and costs. Furthermore, the FDA has adopted organ-on-a-chip data to support a new drug clinical trial (NCT04658472) (Querol et al., 2023; Xiang et al., 2024). Advances in biotechnology coupled with the establishment of standards are driving the translation of PDOs from a research tool into routine clinical practice.

However, organoids also face limitations in precision medicine. Currently, constructing composite organoids that contain both endocrine and exocrine tissues remains a significant challenge (Chen et al., 2024). Additionally, there are currently no globally unified standard protocols for the culture medium formulations and growth conditions of patient-derived organoids (PDOs). This lack of harmonized standards hinders direct comparison and integration of data across different laboratories or medical centers, thereby impeding large-scale clinical application.

## 4 Conclusion and perspectives

With the evolution of the precision medicine paradigm, PDOs are reshaping the decision-making framework for pancreatic cancer treatment by integrating genomic features, dynamic drug sensitivity testing, and clinical prognostic data. In the future, it is essential to further explore the interdisciplinary integration of PDOs with liquid biopsies and artificial intelligence predictive models to achieve comprehensive optimization of treatment strategies.

However, there remains significant potential for further advancements in pancreatic organoid and spheroids research. Firstly, the tumor microbiome has the potential to impact the response of pancreatic cancer to drug therapy (LaCourse et al., 2022). As such, there is a need for the construction of pancreatic 3D co-cultured with microbes in the future to better guide drug therapy for pancreatic tumors. Moreover, there currently lacks pancreatic models for certain benign pancreatic diseases, such as chronic pancreatitis. Yet, the integration of gene editing technologies, such as CRISPR, with 3D cultures is poised to facilitate the development of models capable of predicting *in vivo* drug responses for a broader spectrum of diseases in the future (Tuveson and Clevers, 2019).
